# Cysticercosis Mimicking Fibroadenoma of the Breast in a Young Female: A Case Report From North Central Nigeria

**DOI:** 10.7759/cureus.38141

**Published:** 2023-04-26

**Authors:** Kevin N Ezike, Ijeoma A Okwudire-Ejeh, Bamnan C Dallang, Reuben Ndaiya

**Affiliations:** 1 Anatomic Pathology and Forensic Medicine, Nile University of Nigeria, Abuja, NGA; 2 Anatomic Pathology and Forensic Medicine, Asokoro District Hospital, Abuja, NGA; 3 General Practice, Machanda Hospital, Karu, NGA

**Keywords:** taenia solium, cysticerci, cysticercus, breast, cysticercosis

## Abstract

The parasitic infestation, cysticercosis, arises when humans are infested with the larvae (cysticerci) of the pork tapeworm, *Taenia solium* (*T. solium*). Epidemiologically, cysticercosis presents a worldwide distribution due in part to endemicity in developing countries in Latin America, Asia, and sub-Saharan Africa, and increased migration from these countries to more developed countries in Europe and North America. Cysticercosis may be asymptomatic or may manifest clinical symptoms and signs depending on which part of the body cysticerci are found, including skeletal and heart muscle, skin, subcutaneous tissues, the lungs, liver, the central nervous system (CNS), and less commonly, the oral mucosa and breast.

We report a case of a mass in the left breast in an 11-year-old Nigerian girl, which was diagnosed clinically and on ultrasonography as fibroadenoma but was confirmed on histology to be cysticercosis.

Cysticercosis should be included in the differential diagnoses of breast lumps in persons of all ages and sex, especially in endemic areas and in places with significant immigration from endemic areas.

## Introduction

Cysticercosis in humans is a parasitic infestation resulting from the presence of the larval form, Cysticercus cellulosae, of the pork tapeworm, *Taenia solium* (*T. solium*) [1,2]. *T. solium* is a species of parasitic tapeworm belonging to the Taeniidae family (Phylum: Platyhelminthes, Class: Cestoda, Subclass: Eucestoda, Order: Cyclophyllidea) [3]. Humans are the definitive hosts of *T. solium* while swine/pigs are usually the intermediate hosts. Humans can however become accidental intermediate hosts following ingestion of infectious eggs of the parasite and it is in this scenario that cysticercosis develops.

Human cysticercosis is found worldwide, especially in areas where pig cysticercosis is common. Both taeniasis and cysticercosis are most often found in rural areas of developing countries with poor sanitation, where pigs roam freely and eat human faeces such as in parts of Latin America, Asia, and sub-Saharan Africa [2]. Taeniasis and cysticercosis are rare among persons who live in countries where pigs are not raised and in countries where pigs do not have contact with human faeces. However, cysticercosis is reemerging in developed countries due to increased immigration, travel, and commerce [2,4].

The symptoms of cysticercosis vary depending upon the location and number of cysticerci as well as the host's response to the parasite [2,4]. Cysticerci may develop in skeletal and heart muscle, skin, subcutaneous tissues, the lungs, the liver, the central nervous system (CNS), and less commonly in other tissues, including the oral mucosa and breast [1]. In most locations, cysticerci cause few symptoms and spontaneously degenerate.

We report a case of cysticercosis of the left breast in an 11-year-old Nigerian girl, which was diagnosed clinically and on ultrasonography as fibroadenoma, highlighting the need for its consideration in the differential diagnosis of breast lumps in endemic areas and in areas with significant immigration from endemic areas.

## Case presentation

An 11-year-old schoolgirl presented with a lump in the upper portion of the left breast for the one-month duration. The lump was painful and mobile, with a recent increase in size. It was not related to previous trauma nor were there swellings at other sites. There were no axillary lymph nodes. She is yet to attain menarche and had no significant medical history. She had no history of consumption of pork but she resides in an area dominated by people of a low socioeconomic status, where open rearing of farm animals including pigs, poultry, goats, and cattle, is still prevalent. A positive family history of breast lump was elicited as her aunt had had an excision of a lump many years before.

Clinical examination revealed a mass at the upper outer quadrant of the left breast that was mobile, tender, and free from the overlying skin and underlying structures. There were no palpable axillary lymph nodes. A breast ultrasound scan showed a hypoechoic mass in the upper outer quadrant of the left breast measuring 2.1 x 1.2cm, with no calcifications and/or axillary lymph node enlargement noted. An assessment of fibroadenoma was made with the recommendation of an excisional biopsy. She then had an excision of the mass finding a mass measuring 2 x 3cm at the 12 o’clock position. The specimen was preserved in 10% neutral buffered formalin and sent for histopathological evaluation.

**Figure 1:**
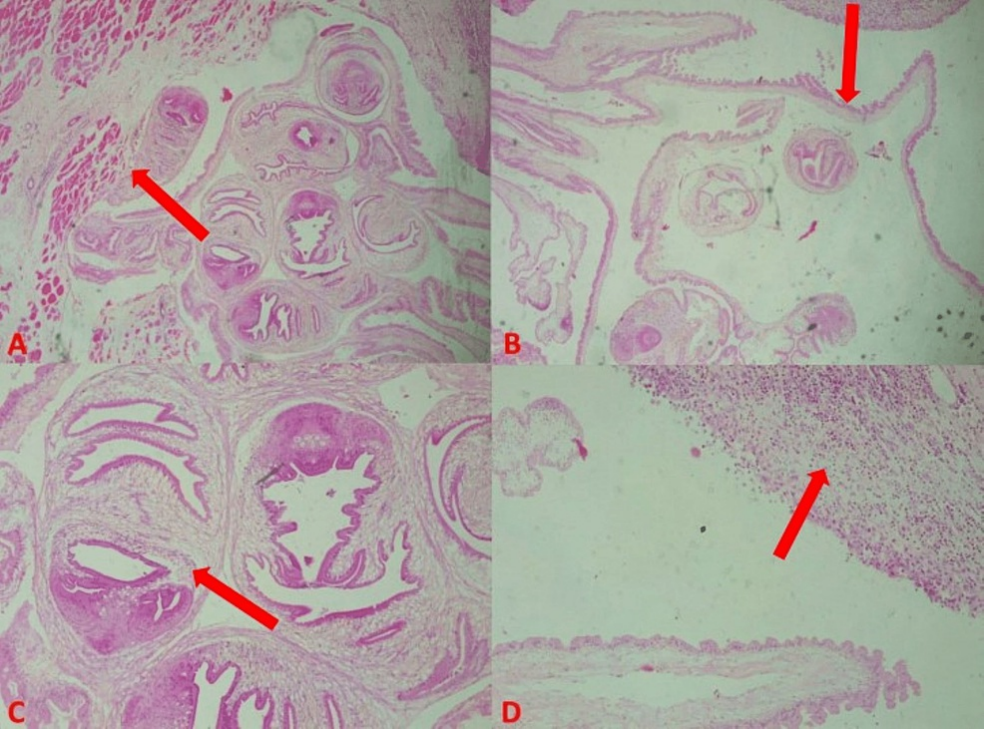
Cysticercosis of the breast (A) Note cyst wall and cysticercus scolex partially embedded in the adjacent skeletal muscle tissue. H&E x40. (B) Note cysticercus body is lined by a double-layered eosinophilic membrane and exhibiting a myxoid matrix. H&E x100. (C) Note cysticercus scolex with hooklets and duct-like invaginations also lined by a double-layered eosinophilic membrane. H&E x200. (D) Note mixed inflammatory cell infiltrates composed predominantly of eosinophils. H&E x100.

The pathological examination revealed gross findings of a circumscribed, greyish-white, firm to fluctuant mass measuring 3.5 x 2.5 x 2.2cm and weighing 10g. The cut section was partially cystic and partially solid. The cyst measured 3cm in its widest diameter and contained clear serous fluid, while the solid component was greyish-white. Histological sections showed a benign cystic lesion composed of a fibrous wall within which is seen the larval form (cysticerci) of the cestode, *T. solium*, having duct-like invaginations lined by a double-layered eosinophilic membrane. The cysticercus is composed of a scolex and body wall that exhibits a myxoid matrix. The cyst wall was partially embedded in the adjacent skeletal muscle tissue. Both the fibrous wall and adjacent skeletal muscle exhibited marked inflammatory response comprising mixed infiltrates of predominantly eosinophils, and also neutrophils, plasma cells, lymphocytes (which formed occasional aggregates with well-developed germinal centres), and scattered epithelioid macrophages (which formed occasional multinucleated, foreign-body type, inflammatory giant cells). No structures reminiscent of breast parenchyma were seen. There was no cytological atypia. A diagnosis of cysticercosis of the left breast was made (Figure 1).

**Figure 2:**
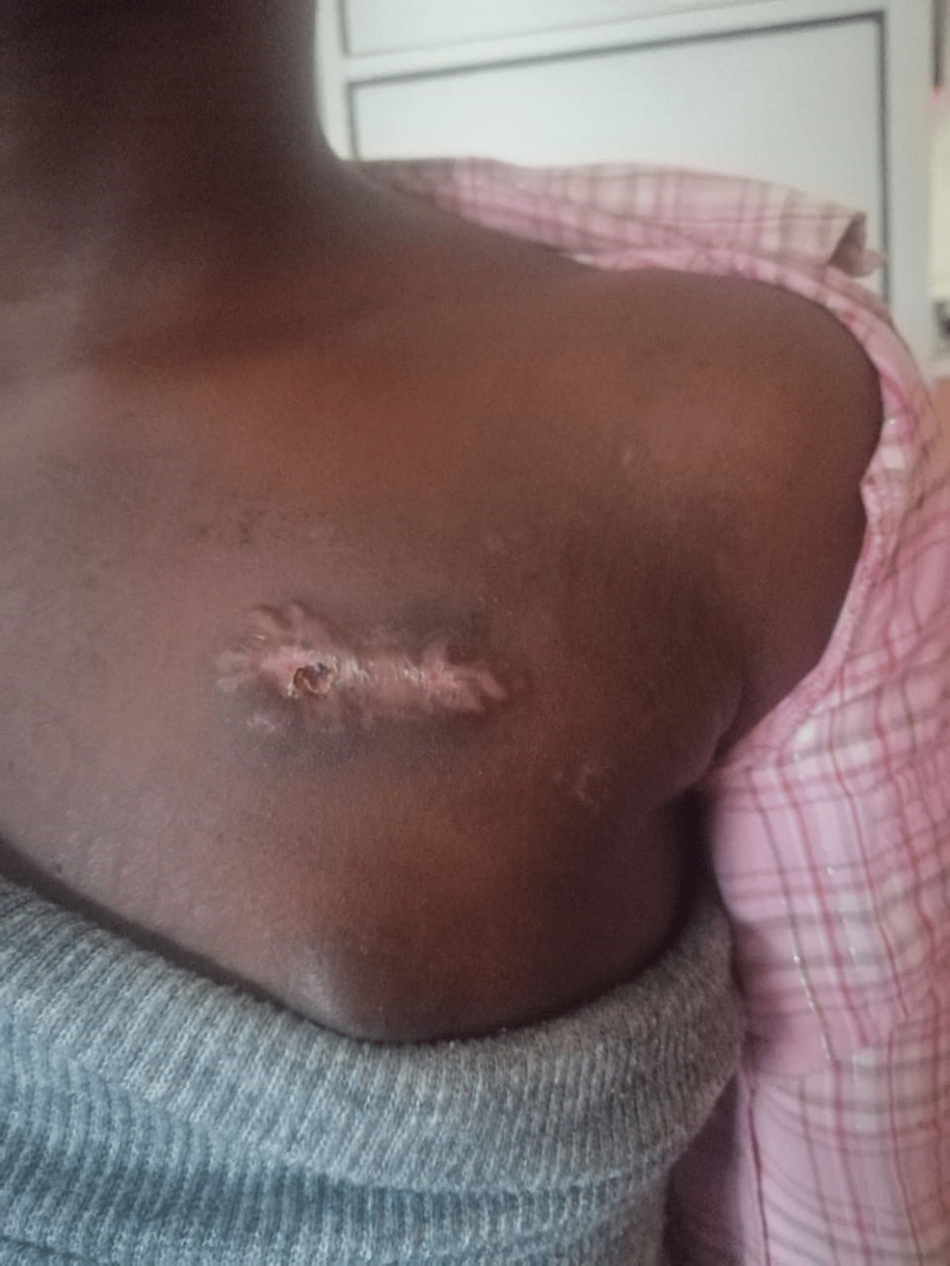
Healed surgical incision site with prominent scar tissue formation

Following excision, post-operative antibiotics, and analgesics were given, and the patient was discharged home the same day in satisfactorily stable condition. On the seventh day of the postoperative review, the incision wound was almost completely healed. The patient was stable, pain-free, and in no obvious distress. However, consequent upon the histopathology report, the anthelminthic, praziquantel, in an oral dosage of 50mg/kg/day for 15 days, was prescribed. Computed tomography (CT) scan was also requested to rule out CNS involvement but the patient is yet to comply due to financial constraints. On the one-month postoperative review, the patient was still stable and pain-free and had suffered no recurrence. The wound site was healed but was developing prominent scar tissue (Figure 2).

## Discussion

Humans are its definitive host but the life cycle of *T. solium* passes through pigs. Pigs ingest eggs and/or gravid proglottids via contaminated food or water and the embryos (oncospheres) of *T. solium *penetrate the gastrointestinal mucosa of the pig with subsequent hematogenous spread to peripheral tissues with the formation of larval cysts - cysticerci. When humans consume undercooked pork, an intestinal tapeworm will be formed, and the life cycle of the worm is completed [1]. Humans get cysticercosis when they swallow eggs by drinking or eating food contaminated with *T. solium* eggs or putting contaminated fingers in their mouths. People who have tapeworm infestations called Taeniasis may also infect themselves with eggs from poor hand hygiene and then develop cysticercosis. This is called autoinfection. People who live with someone who has a tapeworm infection in their intestines have a much higher risk of getting cysticercosis than other people [2]. Although our patient does not eat pork, she was at risk for cysticercosis because she lives in an environment in which pork is reared openly and hence contaminates food and water sources with *T. solium* eggs [1].

Cysticercosis has been declared the number one food-borne infection, affecting about 50 million people globally, and can affect any organ [3]. Common sites of involvement are the subcutaneous tissue, skeletal muscle, heart, and eye. The breast is, however, an unusual site of involvement [1,5-9]. The trend is no different in Nigeria, with only one documented case in a middle-aged female and that, mimicking a carcinoma [10]. To the best of our knowledge, our case is just the second to be reported from Nigeria.

Severe cysticercosis is due to larvae located in human CNS - neurocysticercosis (NCC) [11]. Cysticerci can migrate to the CNS and cause NCC, which is associated with serious neurological and epileptic manifestations. Death can occur suddenly. NCC is typically divided into parenchymal and extraparenchymal diseases. Parenchymal NCC occurs when cysticerci develop within the brain tissue. Extraparynchymal NCC occurs when cysticerci develop in other parts of the nervous system, such as the subarachnoid space, meninges, ventricles, spine, or eyes [2]. The risk of NCC is present in any patient who manifests cysticercosis in any other location, and our patient is no different.

The presenting complaint of cysticercosis of the breast is typically a painless mass. Our patient’s breast mass was, however, associated with pain. While this should raise suspicion of an inflammatory and/or infectious lesion, it should be borne in mind that breast masses whether painful or painless, in the prepubertal female, are non-specific and can be due to many causes. Such causes include normal developmental changes related to menarche like gynecomastia, benign neoplastic lesions like fibroadenoma especially the juvenile type, fibrocystic changes, inflammatory lesions like abscesses, and rarely malignant neoplasms [12].

The pathogenesis of the brisk inflammatory response, seen on histology, in the cyst wall of our patient’s lesion has not been fully elucidated in tissue cysticercosis but literature abounds on its possible mechanism in NCC [1,13]. For the oncosphere of *T. solium *to successfully establish a cyst in the host, it needs to be able to evade the host's immune defences. Possible mechanisms for this include the secretion of prostaglandins by the cysts and inhibition of effective immune responses, including depression of the host’s proliferative responses [13]. When established, these cysticerci may remain viable for years but with time they begin to degenerate and lose their ability to control the host defences [1,13]. When this happens, a strong inflammatory response is elicited leading to the development of a well-defined inflammatory capsule, formed by a cellular inflammatory reaction (composed of plasma cells, lymphocytes, macrophages, and eosinophils) [13]. In NCC, changes around the degenerating cyst include proliferation and activation of astrocytes and microglia, neuronal degeneration and oedema, and perivascular lymphocytic infiltrates of nearby blood vessels [13]. A similar process of persistent residual chronic inflammation and fibrosis in surrounding tissue may account for the scar formation at healing seen in our patient.

## Conclusions

Although there are more common causes, cysticercosis should be included in the differential diagnoses of breast lumps in persons of all ages and sexes, especially in endemic areas and in places with significant immigration from endemic areas. The necessity for histological confirmation of the diagnosis of breast lumps, in general, is also highlighted as the identification of cysticercosis in the breast should raise suspicion of its presence in more sinister sites, including the CNS.

## References

[1] (2023). Cysticercosis (pork tapeworm infection). https://emedicine.medscape.com/article/215589.

[2] (2023). Parasites - cysticercosis. https://www.cdc.gov/parasites/cysticercosis/.

[3] Dixon MA, Winskill P, Harrison WE, Basáñez MG (2021). Taenia solium taeniasis/cysticercosis: from parasite biology and immunology to diagnosis and control. Adv Parasitol.

[4] Symeonidou I, Arsenopoulos K, Tzilves D, Soba B, Gabriël S, Papadopoulos E (2018). Human taeniasis/cysticercosis: a potentially emerging parasitic disease in Europe. Ann Gastroenterol.

[5] Upadhyaya V, Narain D, Sarkar S (2010). Cysticercosis of the breast. J Diagn Med Sonogr.

[6] Attri Attri, N N, Kumari S (2019). Cysticercosis of the breast: a rare finding. J Med Sci Clin Res.

[7] Saigal RK, Sandhu SK, Sidhu PK, Gupta KK (1984). Cysticercosis in Patiala (Punjab). J Postgrad Med.

[8] Amatya BM, Kimula Y (1999). Cysticercosis in Nepal: a histopathologic study of sixty-two cases. Am J Surg Pathol.

[9] Agrawal R (2012). Soft tissue cysticercosis: study of 21 cases. J Clin Diagn Res.

[10] Omonisi AE, Odujoko OO, Aluko JA, Akinyemi HA, Alatishe OI, Omoniyi-Esan GO (2014). Human cysticercosis of the breast mimicking breast cancer: a report of a case from Ile-Ife, Nigeria. Niger J Med.

[11] Bouteille B (2014). Epidemiology of cysticercosis and neurocysticercosis [Article in French]. Med Sante Trop.

[12] Kaneda HJ, Mack J, Kasales CJ, Schetter S (2013). Pediatric and adolescent breast masses: a review of pathophysiology, imaging, diagnosis, and treatment. AJR Am J Roentgenol.

[13] Garcia HH, Nash TE, Del Brutto OH (2014). Clinical symptoms, diagnosis, and treatment of neurocysticercosis. Lancet Neurol.

